# Double Diffusive Magnetohydrodynamic (MHD) Mixed Convective Slip Flow along a Radiating Moving Vertical Flat Plate with Convective Boundary Condition

**DOI:** 10.1371/journal.pone.0109404

**Published:** 2014-10-24

**Authors:** Mohammad M. Rashidi, Neda Kavyani, Shirley Abelman, Mohammed J. Uddin, Navid Freidoonimehr

**Affiliations:** 1 Mechanical Engineering Department, Engineering Faculty of Bu-Ali Sina University, Hamedan, Iran; 2 University of Michigan-Shanghai Jiao Tong University Joint Institute, Shanghai Jiao Tong University, Shanghai, People's Republic of China; 3 Sama Technical and Vocational Training College, Islamic Azad University, Malayer Branch, Malayer, Iran; 4 Centre for Differential Equations, Continuum Mechanics and Applications, School of Computational and Applied Mathematics, University of the Witwatersrand, Johannesburg, South Africa; 5 Department of Mathematics, American International University-Bangladesh, Banani, Dhaka, Bangladesh; 6 Young Researchers & Elite Club, Hamedan Branch, Islamic Azad University, Hamedan, Iran; China University of Mining and Technology, China

## Abstract

In this study combined heat and mass transfer by mixed convective flow along a moving vertical flat plate with hydrodynamic slip and thermal convective boundary condition is investigated. Using similarity variables, the governing nonlinear partial differential equations are converted into a system of coupled nonlinear ordinary differential equations. The transformed equations are then solved using a semi-numerical/analytical method called the differential transform method and results are compared with numerical results. Close agreement is found between the present method and the numerical method. Effects of the controlling parameters, including convective heat transfer, magnetic field, buoyancy ratio, hydrodynamic slip, mixed convective, Prandtl number and Schmidt number are investigated on the dimensionless velocity, temperature and concentration profiles. In addition effects of different parameters on the skin friction factor, 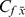, local Nusselt number, 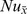, and local Sherwood number 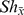 are shown and explained through tables.

## Introduction

Nonlinear equations play an important role in applied mathematics, physics and issues related to engineering due to their role in describing many real world phenomena. The importance of obtaining exact or approximate solutions of nonlinear partial differential equations is still a big problem that compels scientists and engineers to seek different methods for exact or approximate solutions. A variety of numerical and analytical methods have been developed to obtain accurate approximate and analytic solutions for problems. Numerical methods give discontinuous points of a curve and thus it is very time consuming to obtain a complete curve of results. There are also some analytic techniques for nonlinear equations. Some of these analytic methods are Lyapunov's artificial small parameter method [1], 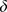-expansion method [2], perturbation techniques [3], [4], variational iteration method (VIM) [5], [6] and homotopy analysis method (HAM) [7], [8].

In recent years semi-numerical/analytical methods have become popular in magnetofluid dynamics research as they provide an alternative to purely numerical methods and require significantly less computational resources. One such method, the differential transform method (DTM) was first introduced by Zhou [9] in electrical circuit theory for solving both linear and nonlinear initial value problems. Developing this method for partial differential equations and obtaining closed form series solutions for linear and nonlinear initial value problems was carried out by Chen and Ho [10] in 1999, and Ayaz [11] applied DTM to the system of differential equations.

The significant advantage of the differential transform method over numerical methods is that it does not require linearization or discretization to be applied to nonlinear differential equations and therefore is not affected by the related errors. Also, DTM does not require a perturbation parameter and also the validity is independent of whether or not there exist small parameters in the considered equation. This method has been adapted in recent years and successfully applied to simulate many multi-physical transport phenomena problems including magnetic liquid film flows [12], mixed convection flow [13], micropolar convection [14].

Magnetohydrodynamics (MHD) is concerned with the mutual interaction of fluid flow and magnetic fields. The fluids being investigated must be electrically conducting and non-magnetic, which limits the fluids to liquid metals, hot ionized gases (plasmas) and strong electrolytes. The use of an external magnetic field is a very important issue in many industrial applications, especially as a mechanism to control material construction. Some important examples of magnetohydrodynamic flow of an electrically conducting fluid past a heated surface are MHD power generators, plasma studies, petroleum industries, cooling of nuclear reactors, the boundary- layer control in aerodynamics, and crystal growth [15], [16]. The goal of the thermal treatment is to cool the material to a desirable temperature before spooling or removing it. As the high temperature material emerges from a furnace or a die, is exposed to the colder ambient, therefore transient conduction process accompanied by surface heat loss is initiated [17]. When high temperatures are encountered in the application areas, the thermal radiation effect becomes very important. High temperature plasmas, cooling of nuclear reactors, liquid metal fluids, and power generation systems are some important applications of radiative heat transfer from a surface plate to conductive fluids. There have been some studies that consider hydromagnetic radiative heat transfer flows. Spreiter and Rizzi [18] studied solar wind radiative magnetohydrodynamics. Nath et al. [19] obtained a set of similarity solutions for radiative-MHD stellar point explosion dynamics using shooting methods. Noor et al. [20] considered MHD free convection thermophoretic flow over a radiate isothermal inclined plate with heat source/sink effect.

Mixed convection flow and heat transfer over a continuously moving surface is applicable to many industrial fields such as hot rolling, paper production, wire drawing, glass fiber production, aerodynamic extrusion of plastic sheets, the boundary-layer along a liquid film, condensation process of metallic plate in a cooling bath and glass, and also in polymer industries [21]. The flow over a continuous material moving through a quiescent fluid is induced by the movement of the solid material and also by thermal buoyancy which will determine the momentum and thermal transport processes [22]. The first study of the flow field due to a surface moving with a constant velocity in a quiescent fluid was undertaken by Sakiadis [23]. Since then, other researchers investigated various aspects of mixed convection problems such as heat and (or) mass transfer, suction/injection, thermal radiation, MHD flow, porous media, slip flows, etc. [24], [25].

In some situations such as the spreading of a liquid on a solid substrate, corner flow and the extrusion of polymer melts from a capillary tube, no slip conditions yield unrealistic behavior and must be replaced by slip conditions especially in applications of microfluidics and nanofluidics [26]–[28]. The difference between the fluid velocity at the wall and the velocity of the wall itself is directly proportional to the shear stress. The proportional factor is called the slip length. The corresponding slip boundary condition is 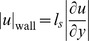, where 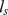 is the slip length [29]. For gaseous flow the slip condition of the velocity and the jump condition of the temperature are 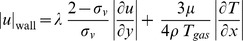 and 
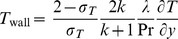
, respectively where 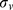 and 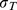 are the tangential momentum coefficient and the temperature accommodation coefficient [30]. Some relevant papers on slip flows are Kim et al. [31], Martin and Boyd [32], Kuznetsov and Nield [33].

The above researchers restricted to either prescribed temperatures or heat flux at the wall or slip. The idea of using convective boundary conditions which are the generalization of isothermal and thermal slip boundary conditions was introduced by Aziz [34] and was followed by Magyari [35] who found an exact solution of Aziz's [34] problem in a compact integral form and Ishak [36] who extended the same problem for a permeable flat plate.

The goal of the present study is to develop similarity transformations via one parameter linear group of transformations and the corresponding similarity solutions for mixed convection flow of viscous incompressible fluid past a moving vertical flat plate with thermal convective and hydrodynamic slip boundary conditions and to solve the transformed coupled ordinary differential equations using the differential transform method. The effects of the Prandtl number
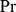
_,_ the Schmidt number 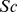, the mixed convective parameter 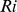, the buoyancy ratio parameter *N*, the radiation parameter *R*, the magnetic field parameter 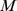 and the slip parameter 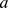 on the flow, heat and mass transfer characteristics are investigated numerically.

In section 2 the geometry of the flow along a moving vertical flat plate under consideration in this problem is modeled with hydrodynamic slip and thermal convective boundary condition assumptions. The system with two independent variables is reduced to one variable equations and a system of nonlinear ordinary differential equations is obtained using a linear group of transformations. Sections 3 and 4 provide methods of solution for the governing equations of the problem, the differential transform method and numerical solution, respectively. In section 5 physical reasons are illustrated for the behavior of the graphs and tables of the problem, and finally inferences are made and conclusions are drawn.

## Nomenclature


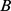
 magnetic field strength


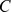
 concentration


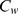
 wall concentration


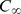
 ambient concentration


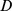
 diffusion coefficient (m^2^/s)


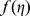
 dimensionless stream function


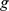
 acceleration due to gravity (m/s^2^)


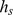
 heat transfer coefficient (W/m^2.^K)

*k* thermal conductivity (W/m.K)


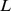
 characteristic length (m)


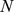
 buoyancy ratio parameter


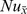
 local Nusselt number


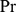
 Prandtl number


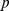
 pressure (N/m^2^)


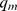
 wall mass flux (kg/s m^2^)


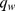
 wall heat flux (W/m^2^)


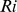
 mixed convective parameter


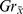
 local Grashof number


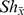
 local Sherwood number


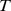
 temperature inside boundary layer (K)


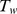
 wall temperature (K)


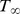
 ambient temperature (K)


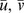
 velocity components along 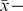 and 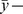
axes (m/s)


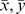
 Cartesian coordinates along and normal to the plate (m)

## Greek Symbols


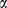
 thermal diffusivity of the porous medium (m^2^/s)


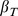
 volumetric thermal expansion coefficient of the base (1/K)


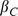
 volumetric solutal expansion coefficient of the base (1/K)


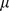
 absolute viscosity of the base fluid (Ns/m^2^)


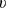
 kinematic viscosity of the fluid (m^2^/s)


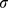
 electric conductivity


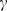
 convective heat transfer parameter


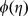
 dimensionless concentration function


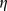
 similarity variable


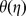
 dimensionless temperature


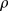
 fluid density (kg/m^3^)


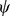
 stream function

## Mathematical Modeling

**Figure 1:**
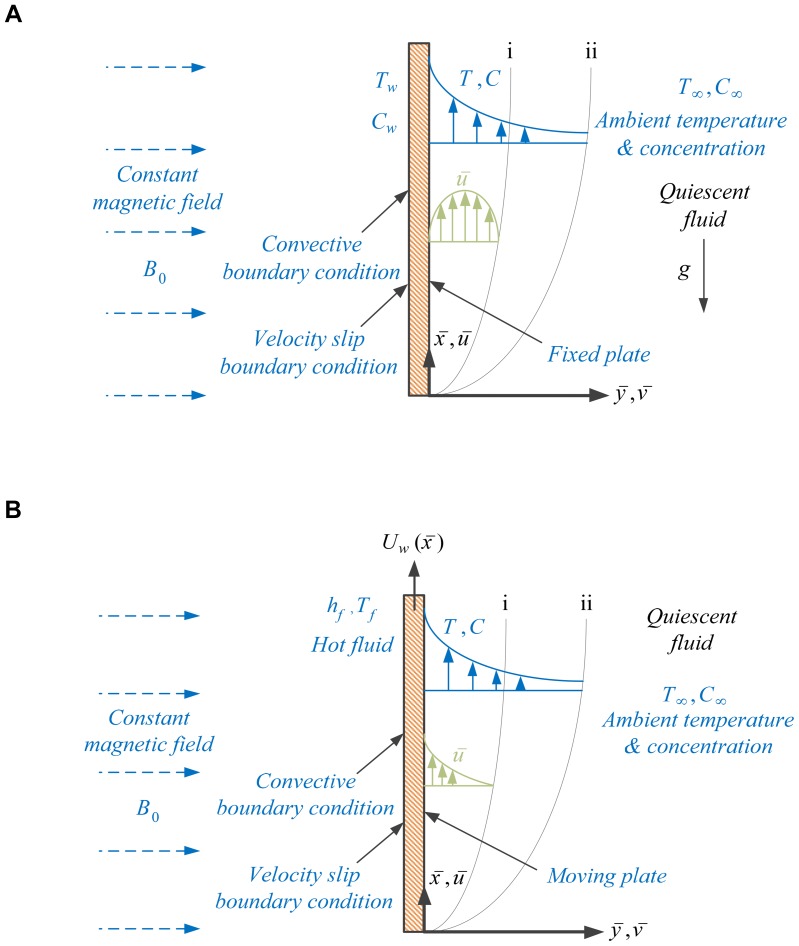
Flow configuration and coordinate system for an assisting flow (a) fixed plate, (b) moving plate.

Figure 1 shows the geometry assumed in this study, along with the rectangular coordinates, 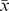 and 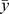, and the corresponding velocity components, 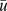 and 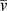 (where i represents momentum, ii represents thermal and concentration boundary-layers and in general thermal and concentration boundary-layer thickness are not the same). The temperature of the ambient fluid is 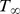, the unknown temperature of the plate is 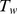 and the left surface of the plate is heated from a hot fluid of temperature 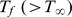 or is cooled from a cooled fluid 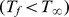 by the process of convection which yields a heat transfer variable coefficient 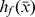. It is also assumed that the ambient fluid is of uniform concentration 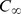, the unknown concentration of the plate is 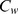. A transverse magnetic field with variable strength 
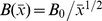
 is applied parallel to the 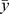 axis, where 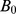 is the constant magnetic field [37]. Variable electric conductivity 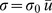 is assumed, where 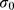 is the constant electric conductivity [37]. The magnetic Reynolds number is assumed to be small and hence the induced magnetic field can be neglected. Fluid properties are invariant except density, which is assumed to vary only in those changes that drive the flow (i.e., the Boussinesq approximation). Under the assumption of boundary-layer approximations, the governing boundary-layer equations in dimensional form are:
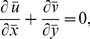
(1)

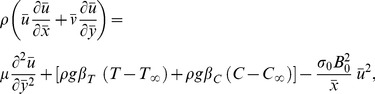
(2)

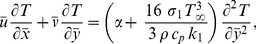
(3)

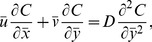
(4)subject to the boundary conditions:
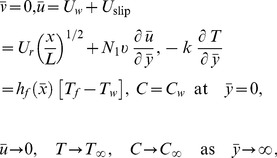
(5)where 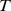 is the temperature, 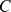 is the concentration, 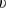 is the kinematic viscosity, 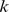 is the thermal conductivity, 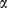 is the thermal diffusivity, 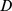 is the mass diffusivity of species of the fluid, 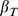 is the volumetric thermal coefficient, 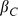 is the volumetric concentration coefficient, 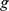 is the acceleration due to gravity, 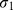 is the Stefan-Boltzmann constant, 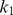 is the Rosseland mean absorption coefficient, 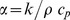 is the thermal diffusivity of the fluid, 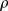 is the density of the fluid, 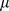 is viscosity, 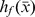 is the heat transfer coefficient and 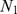 is the velocity slip factor.

### 2.1 Normalization

The following boundary-layer variables are introduced to express Eqs. (1)–(5) in dimensionless form:
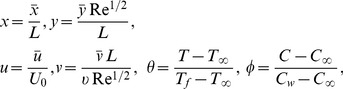
(6)where 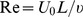 is the Reynolds number based on the characteristic length 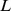 and characteristic velocity 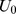. The stream function 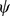 defined as 
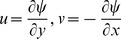
 is substituted into Eqs. (2)–(5) to reduce the number of equations and number of dependent variables. Therefore the following three dimensionless equations are obtained:
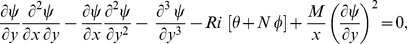
(7)

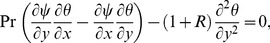
(8)

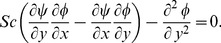
(9)


Here 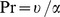 is the Prandtl number, 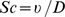 is the Schmidt number, 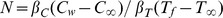 is the buoyancy ratio parameter, 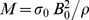 is the magnetic field parameter, 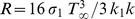 is the radiation parameter, 
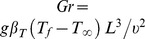
 is the Grashof number, 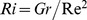 is the mixed convective parameter [38], 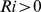 is for aiding buoyancy flow and 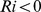 is for opposing buoyancy flow and 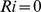 is for purely forced convective flow in which buoyancy effects are not present.

The boundary conditions take the following form:
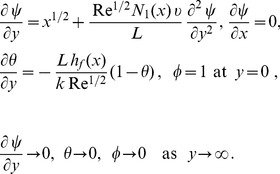
(10)


### 2.2 Application of Linear Group Analysis and Similarity Equations

The transport Eqs. (7)–(10) form a highly coupled nonlinear boundary value problem. Numerical solutions of these equations are complicated and time consuming. In this section the linear group of transformations is imposed on the problem to combine the two independent variables 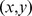 into a single independent variable 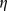 (similarity variable) and reduce Eqs. (7)–(10) into ordinary differential equations with corresponding boundary conditions. For this purpose all independent and dependent variables are scaled as:
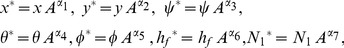
(11)where 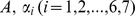 are constants, the values of 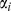 should be chosen such that the form of the Eqs. (7)–(10) is invariant under the transformations. Eqs. (7)–(10) will be invariant if 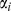 are related by
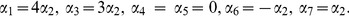
(12)


It is clear from Eqs. (11) and (12) that
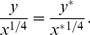
(13)


This combination of variables is invariant under this group of transformations and consequently, is an absolute invariant which are functions having the same form before and after the transformation. This functional form is denoted using
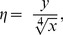
(14)where 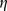 is the similarity independent variable. By the same argument, other absolute invariants are
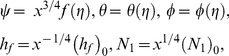
(15)where 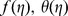 and 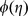 are the dimensionless velocity, temperature, concentration function, 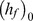 is the constant heat transfer coefficient, 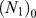 is the constant hydrodynamic slip factor.

Substituting Eqs. (14) and (15) into Eqs. (7)–(9), the following ordinary differential equations are obtained
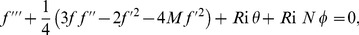
(16)

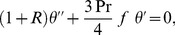
(17)

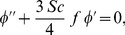
(18)subject to the boundary conditions
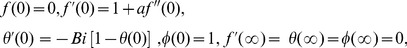
(19)where primes denote differentiation with respect to 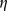. Here 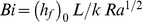 is the Biot number and 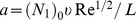 is the hydrodynamic slip parameter. Nondimensionalizing procedure is explained in detail in **Appendix A**.

### 2.3 Quantities of Engineering Interest

The physical parameters of interest in the present problem are the skin friction factor 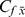, local Nusselt number 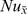 and local Sherwood number 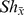 which may be determined, respectively by the following expressions:
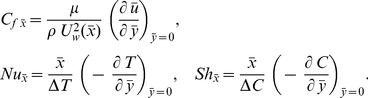
(20)


Using Eqs. (6), (14), (15), we have from Eq. (20)

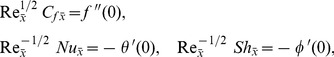
(21)where 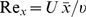 is the local Reynolds number.

## The Differential Transform Method

DTM is employed to obtain semi-analytical/numerical solutions to the well-posed two-point boundary value problem defined by Eqs. (16)–(18) and conditions (19). DTM is an extremely strong technique in finding solutions to magnetohydrodynamic and complex material flow problems. It has also been used very effectively in conjunction with Padé approximants. Rashidi et al. [39] studied transient magnetohydrodynamic flow, heat transfer and entropy generation from a spinning disk using DTM- Padé. To provide a summary of the method, the transformation of the *k* ^th^ derivative of a function in one variable is considered which is defined as:
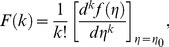
(22)where 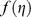 is the original function and 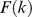 is the transformed function. The differential inverse transform of 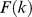 is:
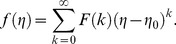
(23)


The concept of the differential transform is derived from a Taylor series expansion and in actual applications the function 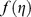 is expressed by a finite series as follows:
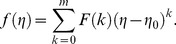
(24)


The value of *m* is decided by convergence of the series coefficients. The fundamental mathematical operations performed by DTM are listed in **Table 1**. Taking differential transforms of Eqs. (16)–(18), the following transformed equations are obtained:
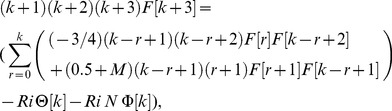
(25)

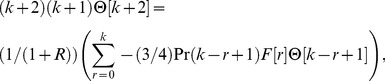
(26)

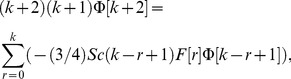
(27)where 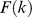, 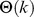 and 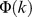 are the differential transform of 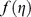, 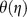 and 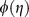, respectively, and the transformed boundary conditions are:
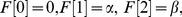
(28)

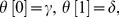
(29)

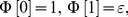
(30)where 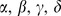 and 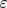 are constants which are computed from the boundary conditions.

**Table 1:** Operations for DTM.

Transformed function	Original function
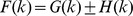	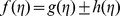
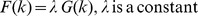	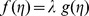
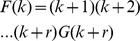	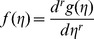
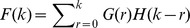	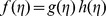
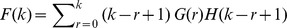	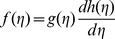
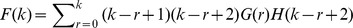	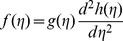

**Figure 2:**
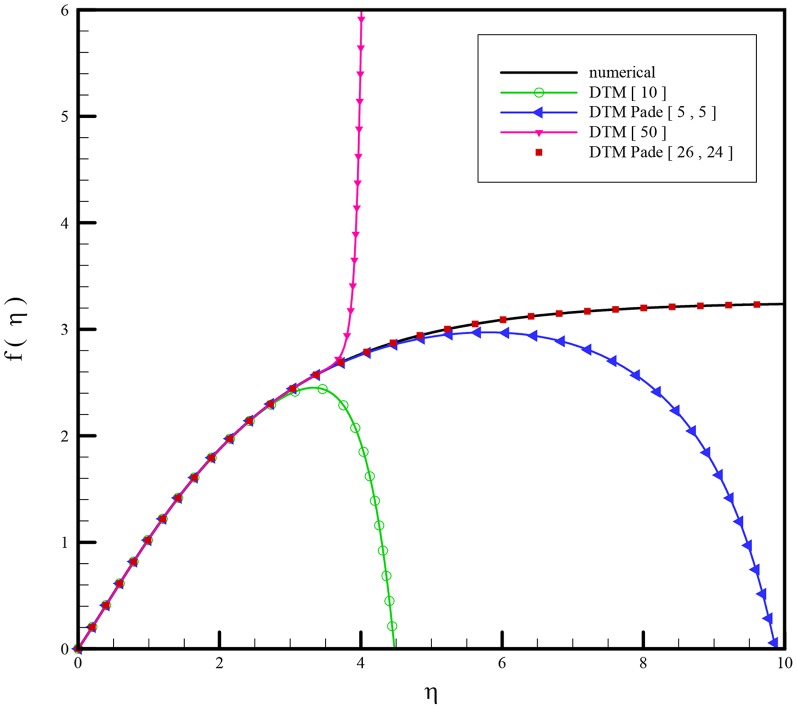
The analytical solution of 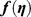 obtained by DTM, DTM-Padé and numerical method for 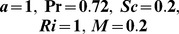 and 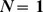
.

Here Páde approximants are applied to the problem to increase the convergence of a given series. As shown in Fig. 2, without using the Páde approximant, the different orders of the DTM solution, cannot satisfy the boundary conditions at infinity. Therefore, it is necessary to use DTM-Páde to provide an effective way to handle boundary value problems with boundary conditions at infinity. See **Appendix B** for a description of the Páde approximant method.

## Numerical Solution

The system of nonlinear differential equations (16)–(18) is solved under the boundary conditions (19). The initial boundary conditions for 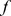 and 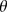 in (19) are unknown in comparison with the case of no-slip and no jump condition of the temperature boundary conditions. Hence, the solution of the system cannot proceed numerically using any standard integration routine. Here, following [40]–[41], a second order numerical technique is adopted. This technique combines the features of the finite difference method and the shooting method and is accurate because it uses central differences.

The semi-infinite integration domain 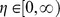 is replaced by a finite domain 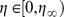 and 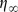 should be chosen sufficiently large so that the numerical solution closely approximates the terminal boundary conditions (19). Here a large enough finite value has been substituted for 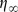, the numerical infinity, to ensure that the solutions are not affected by imposing the asymptotic conditions at a finite distance. The value of 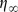 has been kept invariant during the run of the program.

Now a mesh is defined by 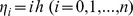, with 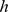 being the mesh size, and Eqs. (16)–(18) are discretized using central difference approximations for the derivatives, the following equations are obtained at the *i^th^* mesh point:
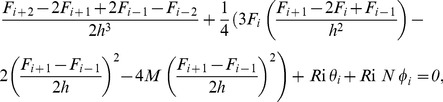
(31)

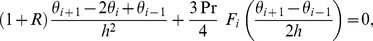
(32)

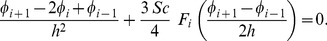
(33)


Note that Eqs. (16)–(18), which are written at the *i^th^* mesh point, the first, second and third derivatives are approximated by central differences centered at *i^th^* mesh point. This scheme ensures that the accuracy of 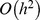 is preserved in the discretization.

Eqs. (31)–(33) are term recurrence relations in 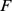, 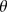 and 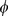. So, in order to start the recursion, besides the values of 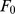, 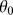 and 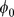, the values of 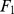, 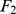, 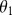 and 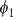 are also required. These values can be obtained by Taylor series expansion near 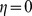 for 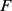, 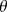 and 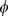, with initial assumptions for the dimensionless functions of 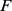 and 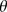.

The values of 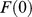, 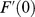, 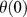 and 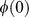 are given as boundary conditions in (19). The values of 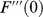, 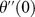 and 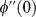 can be obtained directly from Eqs. (10)–(12) and using the initial assumptions. After obtaining the values of 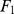, 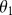 and 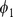, at the next cycle 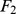, 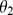 and 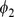 are obtained. The order indicated above is followed for the subsequent cycles. The integration is carried out until the values of 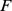, 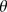 and 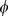 are obtained at all the mesh points.

The three asymptotic boundary conditions (13) and (14) must be satisfied. Initial assumptions are found by applying a shooting method along with the fourth order Runge–Kutta method so as to fulfill the free boundary conditions at 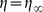 in (19). The guesses can be improved by a suitable zero-finding algorithm, including Newton's method, Broyden's, etc. [42], [43].

## Results and Discussion

A linear group of transformations is used to reduce the two independent variables into one and reduce the governing equations into a system of nonlinear ordinary differential equations with associated boundary conditions. Equations (16)–(18) with boundary conditions (19) were solved analytically using the differential transform method and compared with numerical results. Figure 2 shows the results of comparison and great agreement is seen. Typically, the natural convection is negligible when 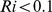, forced convection is negligible when 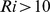, and neither is negligible when 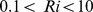. It is useful to note that usually the forced convection is large relative to natural convection except in the case of extremely low forced flow velocities. Here 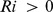 is chosen to have aiding buoyancy flow.

For Biot number smaller than 0.1 the heat conduction inside the body is quicker than the heat convection away from its surface, and temperature gradients are negligible inside of it. Having a Biot number smaller than 0.1 labels a substance as thermally thin, and temperature can be assumed to be constant throughout the materials volume. The opposite is also true: A Biot number greater than 0.1 (a “thermally thick” substance) indicates that one cannot make this assumption, and more complicated heat transfer equations for “transient heat conduction” will be required to describe the time-varying and non-spatially-uniform temperature field within the material body. In this research the case of cooling of the plate 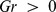 is assumed and also the value of 0.5 is chosen for the Biot number and the thermal radiation number is equal to 1 in all diagrams as the control parameters.

In **Tables 2** **–** **6** effects of some of the parameters on the Skin friction factor, 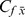, local Nusselt number, 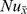, and local Sherwood number, 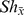 are shown. According to **Table 2**, increasing the buoyancy ratio parameter, *N*, causes 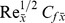, 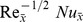 and 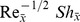 to increase. 
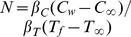
 represents the relative magnitude of species buoyancy and thermal buoyancy forces. For 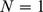 these two forces are of the same magnitude. For 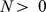, the species buoyancy force is dominant and vice versa for 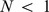. The weaker contribution of the thermal buoyancy force for 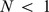 results in a depletion in the local Nusselt number. The stronger contribution of the species buoyancy force for 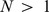 induces enhancement in the local Sherwood number. The momentum field is coupled to the concentration (species) field via the linear species buoyancy force, 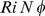, in the momentum Eq. (16). The species field is coupled to the momentum field via the nonlinear term, 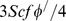, in the species diffusion Eq. (18). Both terms are assistive. The influence of 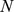 on both species diffusion and momentum diffusion is therefore very strong as observed in the very large magnitudes of skin friction and local Sherwood number in Table 2.

**Table 2:** The Skin friction factor, 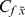, local Nusselt number, 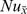 and local Sherwood number, 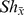 for different values of the buoyancy ratio parameter *N*, at 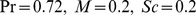, 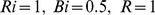 and 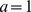.

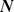	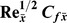	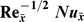	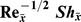
0.1	−0.123402	0.196943	0.220788
1.0	0.118811	0.221377	0.275433
5.0	0.860543	0.259651	0.378371
10.0	1.522606	0.279651	0.444757
50.0	4.644088	0.326977	0.662252
100.0	7.129046	0.346306	0.789132

**Table 3:** The Skin friction factor, 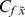, local Nusselt number, 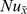 and local Sherwood number, 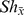 for different values of the mixed convective parameter, 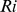, with 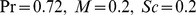, 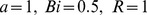 and 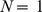.

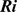	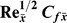	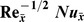	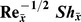
0.2	−0.274494	0.202957	0.300044
0.5	−0.083130	0.203855	0.237817
0.8	0.043656	0.215523	0.262461
1.0	0.118720	0.221333	0.275481
3.0	0.684401	0.251875	0.353565
5.0	1.101863	0.266782	0.398851
7.0	1.451164	0.276702	0.432251
9.0	1.758446	0.284122	0.459204

**Table 4:** The Skin friction factor, 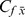, local Nusselt number, 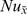 and local Sherwood number, 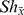 for different values of the hydrodynamic slip parameter, 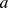, with 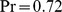, 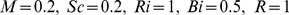 and 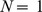.

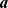	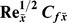	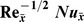	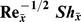
0.0	0.297625	0.218942	0.271244
0.2	0.229317	0.219876	0.272890
0.4	0.186188	0.220452	0.273911
0.6	0.156595	0.220841	0.274604
0.8	0.135608	0.221122	0.275104
1.0	0.118720	0.221333	0.275481
2.0	0.073893	0.221906	0.276507
4.0	0.042066	0.222307	0.277227
6.0	0.029397	0.222465	0.277512
8.0	0.022592	0.222550	0.277665
10.0	0.018345	0.222602	0.277760

**Table 5:** The Skin friction factor, 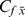, local Nusselt number, 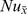 and local Sherwood number, 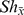 for different values of the Biot number, 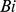, with 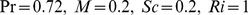, 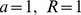 and 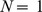.

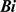	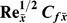	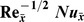	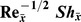
0.1	0.038440	0.079168	0.264176
0.2	0.069660	0.131835	0.268651
0.3	0.091037	0.169820	0.271656
0.4	0.106703	0.198650	0.273830
0.5	0.118721	0.221334	0.275482
0.6	0.128250	0.239673	0.276782
0.7	0.136001	0.254820	0.277833
0.8	0.142435	0.267549	0.278702
0.9	0.147865	0.278401	0.279432
1.0	0.152510	0.287765	0.280055

**Table 6:** The Skin friction factor, 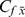, local Nusselt number, 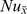 and local Sherwood number, 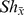 for different values of the Schmidt number, 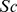, with 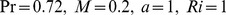, 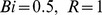 and 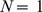.

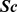	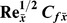	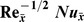	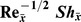
0.22	0.114704	0.220421	0.289944
0.30	0.101054	0.217298	0.342816
0.60	0.068403	0.210346	0.498874
0.66	0.063793	0.209485	0.525015
0.78	0.055692	0.208058	0.573872

In **Table 3**, increasing the mixed convective parameter is observed to strongly increase skin friction and accelerate the boundary-layer flow. Only *Ri*>0 is considered corresponding to *buoyancy-assisted* flow. In the range 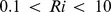 both free (natural) and forced convection modes contribute. For *Ri>10*, forced convection effects are negated and for 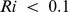 free convection effects vanish. Evidently at low 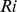 values, buoyancy has a lesser influence on the flow characteristics and skin friction is found to be negative (flow reversal). With *Ri* exceeding unity any flow reversal is eliminated and the flow is strongly accelerated. Stronger buoyancy effects therefore act to stabilize the flow and aid momentum development. This simultaneously encourages more efficient diffusion of heat and species resulting in a marked increase in heat and mass transfer rates (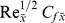, 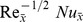) at the plate.

In **Table 4** increasing the value of hydrodynamic slip parameter, 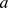, decreases 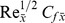
but weakly increases 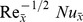 and 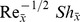. Momentum slip is simulated in the wall velocity boundary condition given in Eq. (19). Increasing momentum slip causes a reduction in the penetration of the stagnant surface through the boundary-layer. This serves to enhance momentum boundary-layer thickness since the flow is decelerated with increasing slip so that skin friction is lowered.


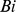
 arises in the wall temperature gradient boundary condition in Eq. (19). As 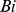 increases from 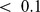 (thermally thin case) to 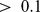 (thermally thick case) the rate of thermal conduction heat transfer inside the plate becomes dramatically lower than the heat convection away from its surface, and temperature gradients are increased at the plate. The influence on the flow is to accelerate it. Skin friction is elevated and therefore momentum boundary-layer thickness decreased. As shown in **Table 5**, an increase in the value of Biot number, 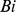, increases 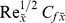, 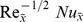 and 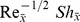.

**Table 6** demonstrates that an increase in Schmidt number, 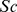, decreases both 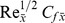 and 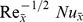, whereas it increases 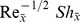. Schmidt number is the ratio of viscous diffusion to molecular (species) diffusion. For 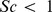, molecular diffusion rate exceeds the momentum diffusion rate and vice versa for 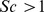. Sub-unity values of Schmidt number will therefore result in a deceleration in the flow (reduced skin friction), which will also decrease thermal diffusion rates. Conversely mass transfer will be accentuated in the regime with increasing 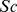 values.

**Figure 3:**
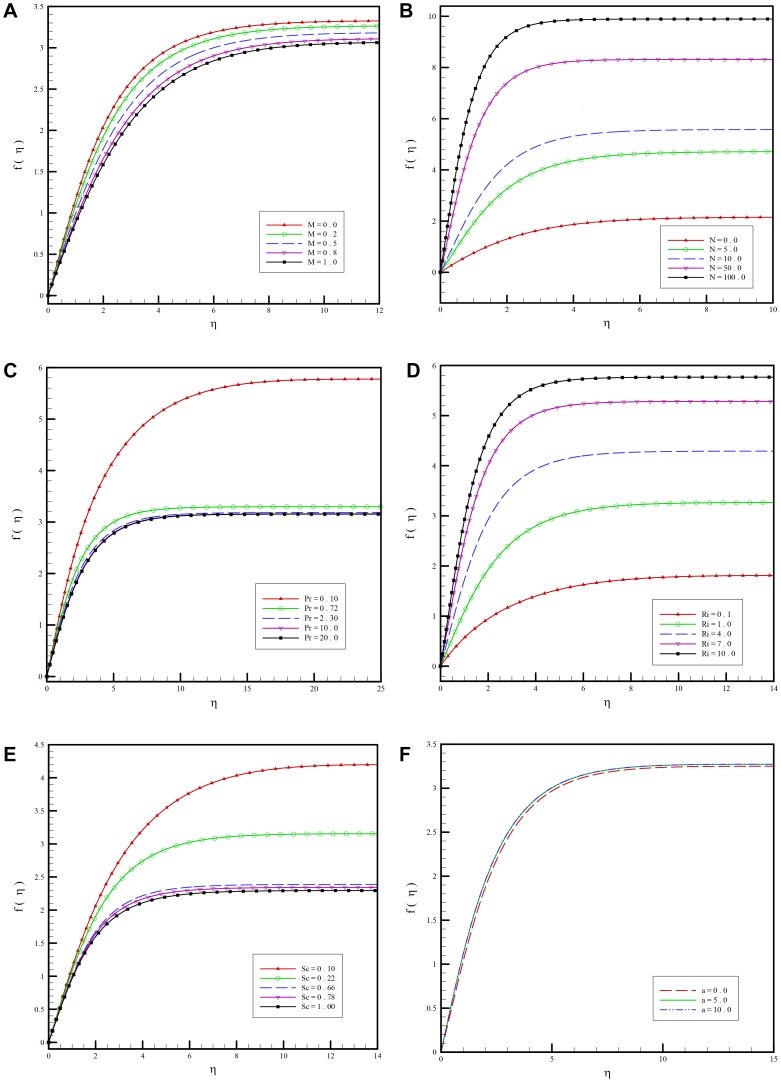
a. Variation of the dimensionless stream function for various values of magnetic field parameter 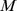 versus 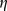 when 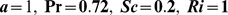 and 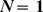. b. Variation of the dimensionless stream function for various values of buoyancy ration parameter 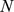 versus 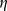 when 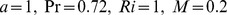 and 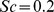. c. Variation of the dimensionless stream function for various values of Prandtl number 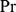 versus 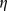 when 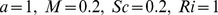 and 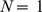. d. Variation of the dimensionless stream function for various values of mixed convective parameter 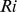 versus 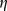 when 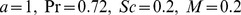 and 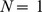. e. Variation of the dimensionless stream function for various values of Schmidt number 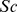 versus 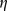 when 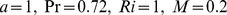 and 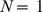. f. Variation of the dimensionless stream function for various values of first order slip parameters 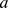 versus 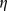 when 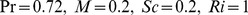 and 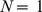.

In **Fig. 2** results using different orders of DTM and DTM-Páde are compared with those of the numerical method. Very good agreement is observed for DTM-Páde and the numerical method. In figures **3. a**–**3. f** effects of different parameters are investigated on the flow regime and the following results are observed:

- **Fig. 3. a** shows how the flow responds to change in the magnetic field. Increasing magnetic interaction number 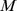 from purely hydrodynamic case 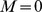 to higher values of 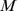, gives rise to a strong deceleration in the flow. Presence of a magnetic field in an electrically conducting fluid introduces a Lorentz force which acts against the flow in the case that magnetic field is applied in the normal direction as considered in the present problem. The described type of resistive force tends to slow down the flow field.
- A positive rise in 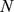 induces an increase in the flow along the plate as seen in **Fig. 3. b.**
- There is a clear decrease in the velocity values at the wall accompanying a rise in Prandtl number because the flow is decelerated. Fluids with higher Prandtl numbers will therefore possess higher viscosities (and lower thermal conductivities) which means that such fluids flow slower than lower 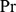 fluids (**Fig. 3. c**). As a result the velocity will be decreased substantially with increasing Prandtl number.
- According to **Fig. 3. d**, a strong mixed convective parameter has a significant acceleration effect on the boundary-layer flow.
- Through changing the values of 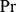 and 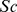, the thermal and species diffusion regions change. As illustrated in **Fig. 3. e**, the dimensionless stream function 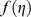 decreases as a result of increasing Schmidt number.
- **Fig. 3. f**, results from comparing the flow in the presence of slip and no slip boundary condition. The change in profiles for different values of 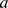 is not so much. In fact 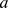 influences the flow of the liquid past the moving plate and the amount of slip 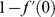 increases monotonically with 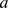 from the no-slip situation of 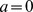 and towards full slip as 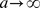. In the limiting case the frictional resistance between the cooling liquid and the moving plate is eliminated, and the moving plate no longer imposes any motion of the cooling liquid.

**Figure 4:**
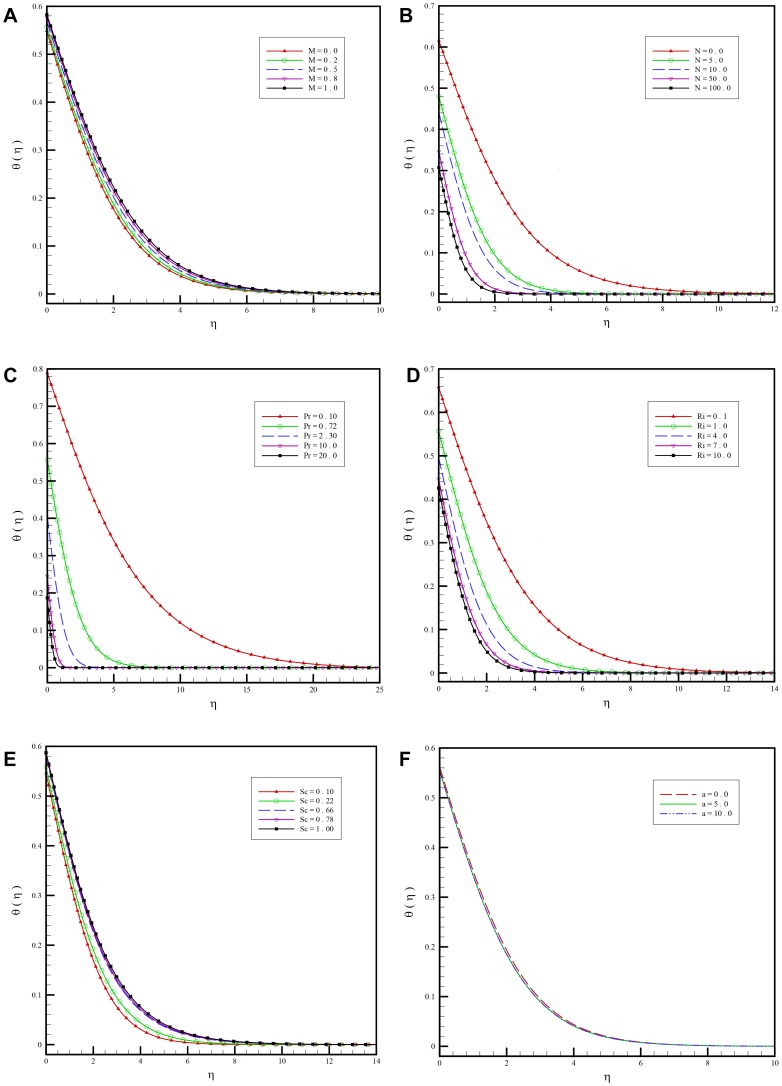
a. Variation of the dimensionless temperature for various values of magnetic field parameter 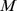 versus 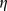 when 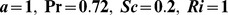 and 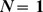. b. Variation of the dimensionless temperature for various values of buoyancy ration parameter 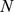 versus 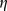 when 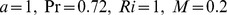 and 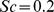. c. Variation of the dimensionless temperature for various values of Prandtl number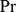 versus 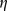 when 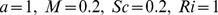 and 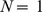. d. Variation of dimensionless temperature for various values of mixed convective parameter 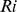 versus 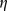 when 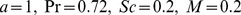 and 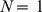. e.-Variation of the dimensionless temperature for various values of Schmidt number 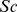 versus 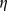 when 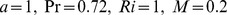 and 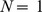. f. Variation of the dimensionless temperature for various values of first order slip parameters 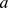 versus 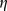 when 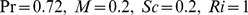 and 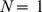.

Figures **4. a**–**4. f** show how heat transfer is influenced by different parameters:

- The magnetic field increases the temperature of the fluid inside the boundary-layer as a result of excess heating and consequently decreases in the heat flux, as shown in **Fig. 4. a**.
- A positive rise in 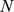 causes the temperature to decrease as seen in **Fig. 4. b**.
- **Fig. 4. c.** depicts the effects of the Prandtl number 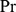 on the temperature profiles 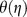. Prandtl number shows the ratio of momentum diffusivity to thermal diffusivity. The figure reveals that an increase in the Prandtl number 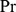
_,_ results in a decrease in the temperature distribution at a particular point of the flow region. The lowest temperatures correspond to the highest value of Prandtl number. No temperature overshoot is observed. The increase in the Prandtl number means a slow rate of thermal diffusion. Larger 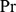 values imply a thinner thermal boundary-layer thickness and more uniform temperature distributions across the boundary-layer. Smaller 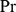 fluids have higher thermal conductivities so that heat can diffuse away from the vertical surface faster than for higher 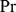 fluids (thicker boundary-layers).
- According to **Fig. 4. d**, temperature decreases by increasing the value of the Richardson number.
- Temperature continuously increases with increasing Schmidt number as depicted in **Fig. 4. e**.
- In **Fig. 4. f**. changing the slip parameter 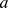 does not affect temperature profiles much.

**Figure 5:**
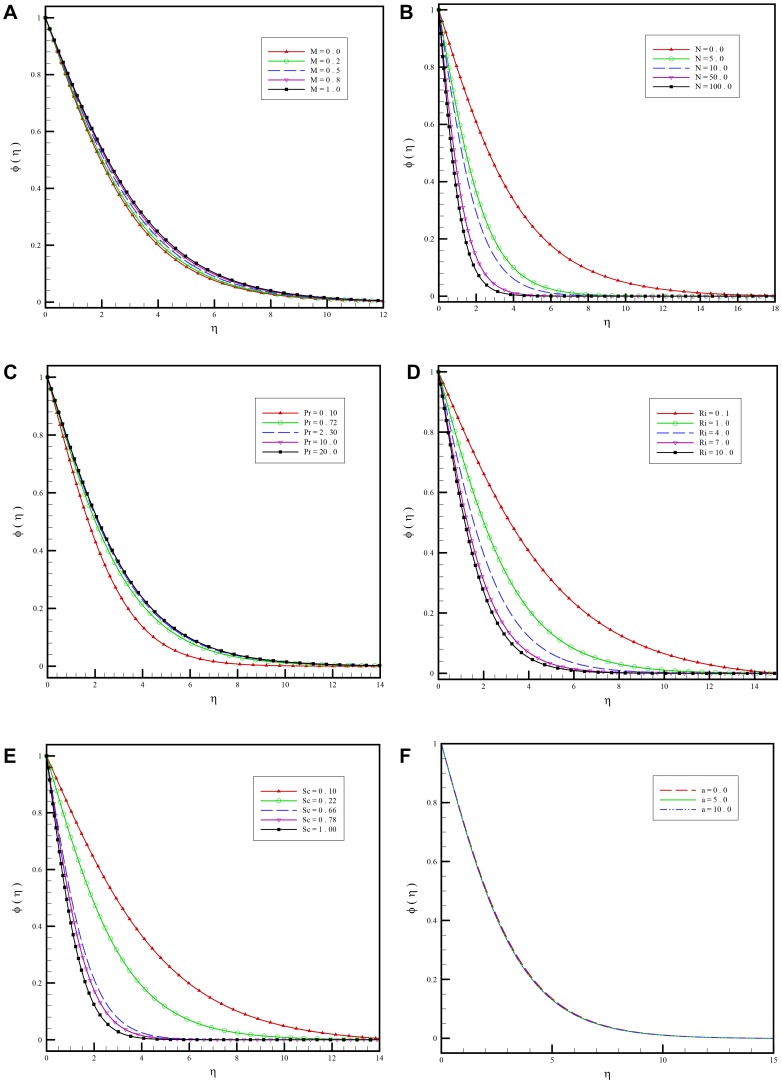
a. Variation of the dimensionless concentration function for various values of magnetic field parameter 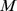 versus 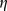 when 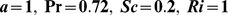 and 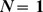. b. Variation of the dimensionless concentration function for various values of buoyancy ration parameter 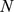 versus 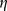 when 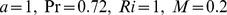 and 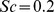. c. Variation of the dimensionless concentration function for various values of Prandtl number 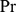 versus 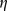 when 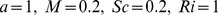 and 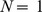. d. Variation of the dimensionless concentration function for various values of mixed convective parameter 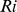 versus 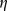 when 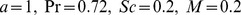 and 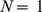. e. Variation of the dimensionless concentration function for various values of Schmidt number 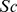 versus 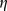 when 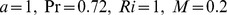 and 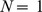. f. Variation of the dimensionless concentration function for various values of first order slip parameters 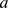 versus 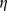 when 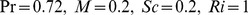 and 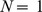.

Figures **5. a**–**5. f** show how the concentration profiles vary through changing different parameters entering into the problem.

- According to **Fig. 5. a** concentrations increase by increasing the value of 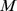.
- Concentration decreases by a positive rise in 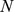 as seen in **Fig. 5. b.**
- **Fig. 5. c** shows the response of the dimensionless concentration function through the boundary-layer regime to Prandtl number 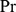.
- The dimensionless concentration function 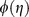 as shown in **Fig. 5. d** is adversely affected through increasing the mixed convective parameter 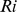. According to **Figs. 4. d.** and **5. d.** both temperature and concentration profiles descend smoothly from the maximum value at the wall to zero in the free stream. Here the value of the buoyancy ratio parameter is unity, 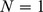 which indicates that the thermal and concentration (species diffusion) buoyancy forces are of the same order of magnitude.
- **Fig. 5. e.** indicates that concentration 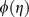 is reduced continuously throughout the boundary-layer with increasing the value of 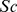. Schmidt number measures the relative effectiveness of momentum and mass transport by diffusion. Larger values of 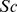 are equivalent to reducing the chemical molecular diffusivity i.e. less diffusion therefore takes place by mass transport.
- **Fig 5. f.** shows that changing the value of the slip parameter 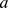 has little influence on the concentration profiles.

## Conclusions

In this study, combined heat and mass transfer of the flow along a moving vertical flat plate with hydrodynamic slip and thermal convective boundary condition was considered. In order to reduce the two independent variables into one and hence to reduce the governing equations into a system of nonlinear ordinary differential equations, a linear group of transformations was used. The obtained equations were solved analytically using the differential transform method. The results were verified by results taken from the numerical method and excellent agreement was observed. The effects of different parameters on the skin friction factor, 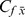, local Nusselt number, 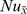, and local Sherwood number 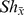 were shown and explained through tables and also changes of dimensionless flow and heat and mass transfer rates due to changes in some parameters were analyzed and presented graphically.

## Appendix A

The governing boundary-layer equations in dimensional form are:
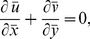
(a1)

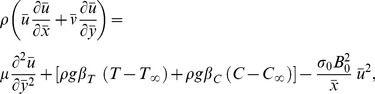
(a2)

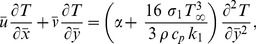
(a3)

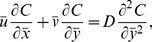
(a4)subject to the boundary conditions:
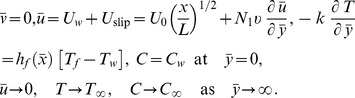
(a5)


Using the following boundary-layer variables:
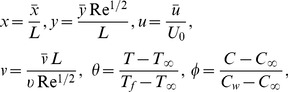
(a6)the following equations are obtained for Eqs. (a2)–(a4):
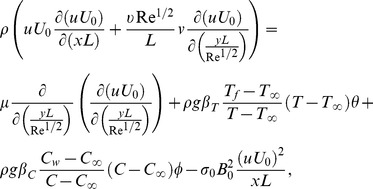
(a7)

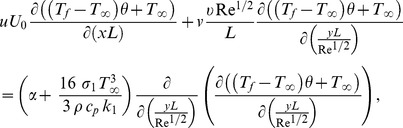
(a8)

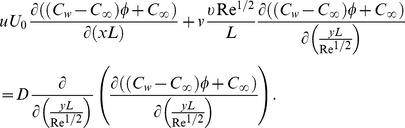
(a9)


After simplifying the equations and dividing Eq. (a7) by 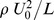, Eq. (a8) by 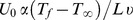, Eq. (a9) by 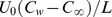, and using the definitions for 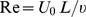, 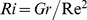, 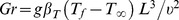, 
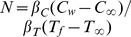
, 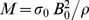, 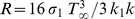, 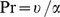, and 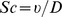 following equations are obtained:
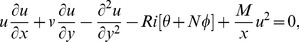
(a10)

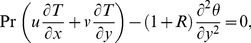
(a11)

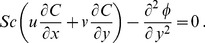
(a12)


The stream function 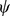 defined as 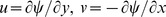 is substituted into Eqs. (a10)–(a12) to reduce the number of equations and number of dependent variables, therefore the following three dimensionless equations are obtained:
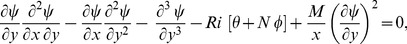
(a13)

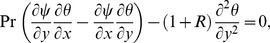
(a14)

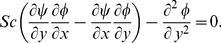
(a15)


The boundary conditions take the following form:
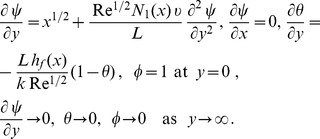
(a16)


All independent and dependent variables are scaled as:
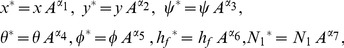
(a17)where 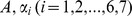 are constants. The values of 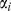 should be chosen such that the form of the Eqs. (a13)–(a15) is invariant under the transformations by substituting the above variables:
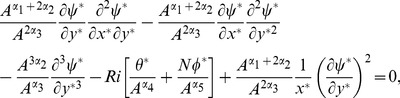
(a18)

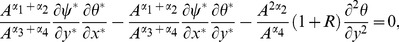
(a19)

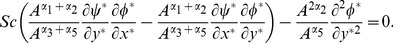
(a20)


Boundary conditions:
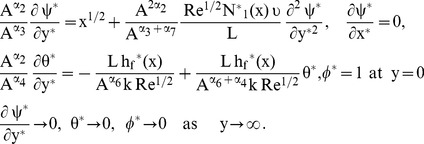
(a21)


Obviously Eqs. (a18)–(a20) will be invariant if 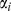 are related by
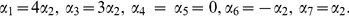
(a22)


Using the similarity independent variable 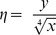 and other absolute invariants such as dimensionless velocity, temperature, concentration function as follows:
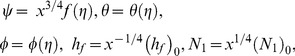
(a23)the following equations are obtained:
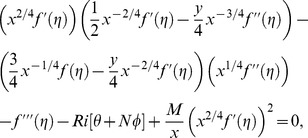
(a24)

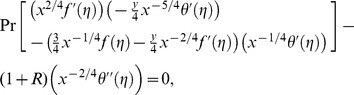
(a25)

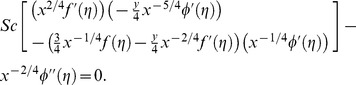
(a26)


After simplifying the above equations, Eqs. (16)–(18) are obtained.

## Appendix B

Suppose that a power series 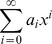 is given, which represents a function 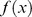, such that:
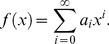
(b1)


The Páde approximant is a rational fraction and the notation for such a Padé approximant is:
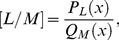
(b2)where 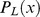 is a polynomial of degree at most 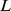 and 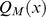 is a polynomial of degree at most 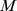. Therefore:
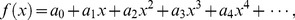
(b3)

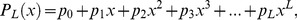
(b4)

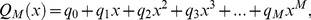
(b5)where in Eq. (b2) there are 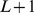 numerator coefficients and 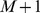 denominator coefficients. Since the numerator and denominator can be multiplied by a constant and 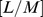 left unchanged, the following normalization condition is imposed
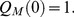
(b6)


So there are 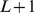 independent numerator coefficients and 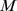 independent denominator coefficients, which make 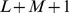 unknown coefficients in all. This number suggests that normally the 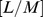 ought to fit the power series Eq. (b1) through the orders 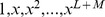. Based on conditions given in [44], [45], 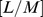 approximation is uniquely determined. In the notation of formal power series:
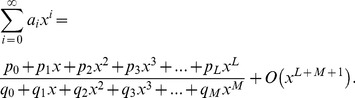
(b7)


By cross-multiplying Eq. (b7), one obtains:
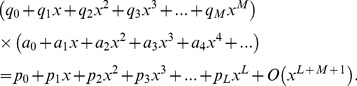
(b8)


From Eq. (b8) the following set of linear equations is obtained
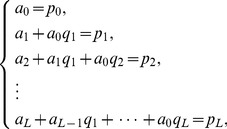
(b9)and
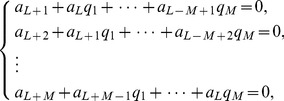
(b10)where 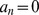 for 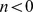 and 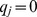 for 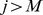. Equations (b9) and (b10) can be solved directly provided they are non-singular.
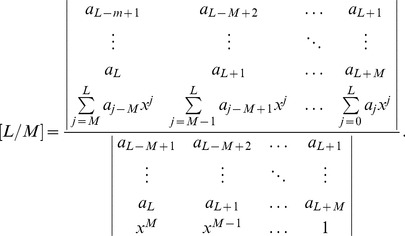
(b11)


If the lower index on a sum exceeds the upper, the sum is replaced by zero. Alternate forms are:
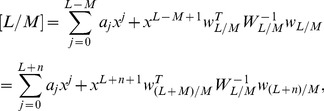
(b12)for
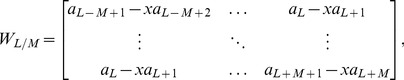
(b13)

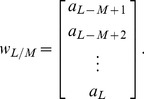
(b14)


The construction of 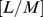 approximants involves only algebraic operations [44], [45]. Each choice of 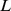, degree of the numerator and 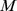, degree of the denominator, leads to an approximant. How to direct the choice in order to obtain the best approximant is the major difficulty in applying the technique, which necessitates the need for a criterion for the choice depending on the s*hape* of the solution. A criterion which has worked well here is the choice of 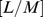 approximants such that 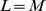
_._ The approximants are constructed using **MATHEMATICA** software.
